# An integrated simulation–optimization framework for assessing environmental flows in rivers

**DOI:** 10.1007/s10661-022-10908-w

**Published:** 2023-01-12

**Authors:** Mahdi Sedighkia, Nasrin badrzadeh, Zeynab Fathi, Asghar Abdoli, Bithin Datta

**Affiliations:** 1 Australian National University, Canberra, Australia; 2 Tarbiat Modares University, Tehran, Iran; 3 University of Kurdistan, Sanandaj, Iran; 4 James Cook University, Townsville, Australia

**Keywords:** Environmental flow, Optimization, Physical habitats, Macroinvertebrates population, Ground water level

## Abstract

The present study proposes an integrated simulation–optimization framework to assess environmental flow by mitigating environmental impacts on the surface and ground water resources. The model satisfies water demand using surface water resources (rivers) and ground water resources (wells). The outputs of the ecological simulation blocks of river ecosystem and the ground water level simulation were utilized in a multiobjective optimization model in which six objectives were considered in the optimization model including (1) minimizing losses of water supply (2) minimizing physical fish habitat losses simulated by fuzzy approach (3) minimizing spawning habitat losses (4) minimizing ground water level deterioration simulated by adaptive neuro fuzzy inference system(ANFIS) (5) maximizing macroinvertebrates population simulated by ANFIS (6) minimizing physical macrophytes habitat losses. Based on the results in the case study, ANFIS-based model is robust for simulating key factors such as water quality and macroinvertebrate’s population. The results demonstrate the reliability and robustness of the proposed method to balance environmental requirements and water supply. The optimization model increased the percentage of environmental flow in the drought years considerably. It supplies 69% of water demand in normal years, while the environmental impacts on the river ecosystem are minimized. The proposed model balances the portion of using surface water and ground water in water supply considering environmental impacts on both sources. Using the proposed method is recommendable for optimal environmental management of surface water and ground water in river basin scale.

## Introduction

Rivers ecosystems are threatened due to considerable reduction of available flow (Sedighkia et al., 2017). Hence, the concept of the environmental flow has been defined for increasing available water to mitigate ecological impacts on the river ecosystem (Nestler et al., 2018). In other words, environmental flow regime might guarantee sustainability of river ecosystems. Assessing environmental flow is a challenging research field from several years ago. Due to complexities of assessing environmental flow regime, it is still a fresh research field in the developed and developing countries (Carvajal-Escobar, 2008).

As a general classification, available methods could be classified in four main groups including hydrologic desktop methods or historic flow methods, hydraulic rating methods, habitat simulation methods and holistic methods. Some methods, such as hydrologic desktop methods, are unreliable in assessing environmental flow regimes due to the inability to highlight the regional ecological values (Sedighkia et al., 2017). Using an ecological based method is advantageous due to highlighting regional values. In fact, we face complex ecological challenges in the river basins that might not be manageable by the old methods of the environmental flow assessment such as hydrological desktop methods.

As a review on available ecological based methods of environmental flow assessment, Instream flow incremental methodology IFIM has been developed in the USA as an integrated framework to assess environmental flow regime (Nestler et al., 2018). It is a general framework to assess environmental flow. Developers encouraged the users to have creativity and innovation in the practical applications of the IFIM. The core of this method is physical habitat simulation (Choi et al., 2018; Waddle, 2001). Multivariate methods such as fuzzy logic approach have been proposed for physical habitat simulation in which verbal fuzzy rules of physical habitat suitability are developed based on the combination of fish observations and expert opinions (Muñoz-Mas et al., 2012; Noack et al., 2013). The main advantage of this method is to apply expert opinions in the simulation of physical habitat suitability that has been utilized in recent studies to assess or optimize environmental flow (Sedighkia et al., 2021a). Building block methodology BBM is another robust framework to assess environmental flow regime that might be considered as a holistic method (originally developed by King & Louw, 1998). This method is able to consider many effective factors in the assessment framework of the environmental flow regime including hydraulic, hydrology, water quality, ecology, geomorphology and groundwater. However, using this method might not be easy practically. First, this method is not based on the simulation of the effective parameters that might weaken the applicability of the method. Moreover, the manual of this method is a general guideline on the requirements of each section to assess environmental flow that means it should be set in accordance with the needs of each case study.

Environmental flow assessment and water resource management should be linked for integrated water resources management. The optimization is an important tool for integrated water resources which has been utilized in the control and management of the surface water resources and groundwater resources (Abdulbaki et al., 2017). For example, reservoir operation optimization is a known problem for all water resources engineers as an application of optimization tools (Ahmad et al., 2014). Linear programming (LP), non-linear programming (NLP) and dynamic programming (DP) have been addressed as conventional optimization methods in water resource management (Feng et al., 2019; Li et al., 2020; Zeng et al., 2019). However, the evolutionary algorithms are recommended for complex functions due to high efficiency (Maurya & Singh, 2021). These algorithms might be classified as the classic and new generation algorithms or animal and non-animal inspired algorithms (more details by Jahandideh-Tehrani et al., 2019; Dokeroglu et al., 2019). A long list of evolutionary algorithms has been used in the optimization problems of water resources engineering (e.g., Afshar et al., 2011; Ehteram et al., 2018; Yaseen et al., 2019; Asgari et al., 2016; Sedighkia & Abdoli, 2021). Furthermore, multiobjective evolutionary algorithms could be applied in the multiobjctive problems (e.g., Sedighkia et al., 2021b). In fact, some problems in the water resource management need for minimization or maximization of two or more objective function simultaneously. Applying multiobjective particle swarm optimization or multiobjective genetic algorithm has been recommended in this regard. More details on the application of the mutliobjective optimization in the water resources problems have been reviewed in the literature (e.g., Ferdowsi et al., 2021).

Developing the integrated frameworks for optimizing the environmental flow is essential in which environmental requirements of river ecosystem and water supply sources should be linked. Some limited studies addressed frameworks for optimizing environmental flow in river basins as cited in the previous paragraph. However, developed frameworks were not integrated which means they are not able to address all needed components in optimization of environmental flow. Due to this research gap, this study develops a novel integrated framework for optimizing environmental flow in the catchment scale in which requirements of river habitats, ground water level and water supply are integrated to balance needs of environment and community.

## Methodology and case study

### Overview on the methodology

**Fig. 1:**
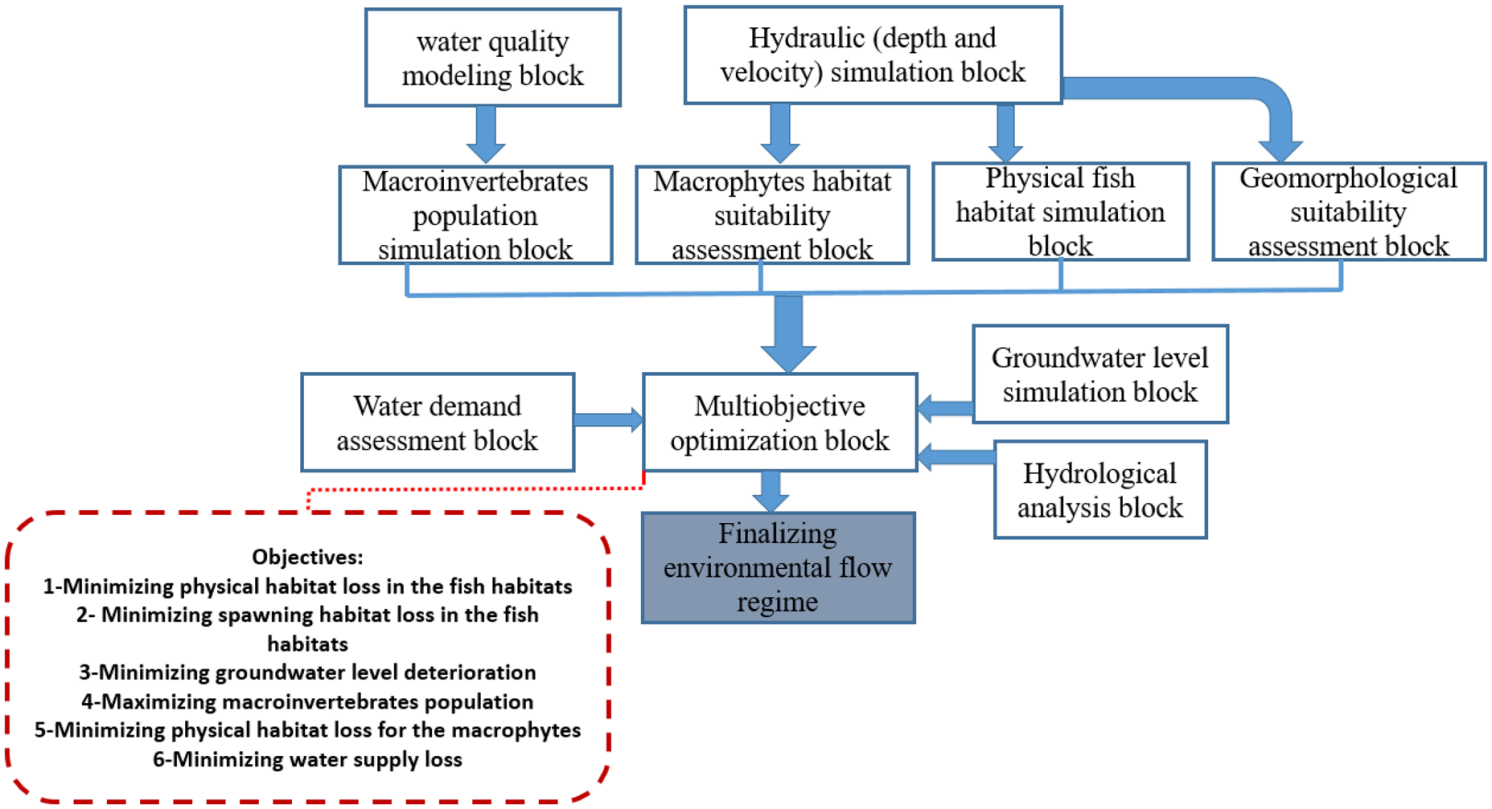
Workflow of the proposed method

The proposed method is a complex framework including different simulation blocks that are utilized in an integrated optimization model. An overview on the workflow of the simulation and optimization blocks methodology (SOBM) might be helpful for the readers. Figure 1 displays flowchart of the proposed framework in which several simulation and assessment blocks are used to generate ecological functions and other required inputs of the optimization model. Finally, a multiobjective optimization model is utilized to optimize environmental flow in the study area. Several objectives are considered in the optimization model as follows:

1. Minimizing physical fish habitats losses
2. Minimizing spawning habitat losses
3. Minimizing ground water level (GWL) deterioration
4. Maximizing macroinvertebrates population
5. Minimizing physical macrophytes habitat losses
6. Minimizing water supply losses

As a short description on the objectives, physical habitat simulation is applied in the assessment of the fish habitats. The purpose of the optimization model is to minimize difference between optimal normalized weighted useable area and maximum normalized weighted useable area (i.e. MNWUA = 1) in each time step. It should be noted that the reproduction is a very important biological activity for different fish species that might guarantee the survival of the fish generations for a long-term period. Hence, it should be considered in the assessment of environmental flow regime. The suitability of the spawning habitats is strongly dependent on the sediment bed transport. One of the aims of the proposed optimization method is to mitigate ground water level deterioration due to interaction between surface water and ground water for supplying water demand. Macroinvertebrates are the important aquatics that might indicate the suitability of the river ecosystem in terms of water quality. Thus, we focused on the maximization of the macroinvertebrates population as one of the objectives in the optimization system macrophytes are the aquatic plant, large enough to be observed by the naked eye. We selected this species as an ecological index to simulate aquatic plant in the assessment of the environmental flow with a focus on the effective physical parameters. Supply of water demand is one of the main objectives in the water resource management that is effective on the environmental flow regime. Hence, maximum water supply was considered as another purpose in the optimization system. Inputs and outputs of the models in the case study will be displayed in the next sections.

### Hydraulic (depth and velocity) simulation block

We applied the HEC-RAS 1D (version 5) to simulate depth and velocity in the cross sections of the representative river reach in the case study. Digital elevation model (DEM) was provided based on the previous river engineering studies in the river reach. Then, cross sections were generated using the HEC-GEORAS (version 10.5) model. Generated cross sections were inserted to the HEC-RAS 1D (version 5) model to simulate depth and velocity. Some measured discharge, depth and velocity in the hydrometric station were utilized for calibration and verification of the hydraulic model in the representative reach. More details regarding the hydraulic simulation have been addressed in the literature (Betsholtz & Nordlöf, 2017).

### Water quality modelling block

Using data-driven models is one of the applicable methods to simulate water quality that have been recommended in the literature due to flexibility, robustness and reliability. Different forms of data driven models have been utilized in this regard. Previous studies demonstrated reliability of the artificial neural networks (ANNs) to simulate water quality (Khan & Chai, 2017; Isiyaka et al., 2019). However, ANNs needed to be improved due to some drawbacks such as acting as a black box (Mijwel, 2018). Neuro fuzzy inference systems (NFSs) are considerably robust to simulate or forecast the response of the complex systems in which several inputs should be considered (Salleh et al., 2017). Due to complexities of the water quality modelling, adaptive neuro fuzzy inference systems (ANFISs) have been applied in this regard (e.g., Khan & Chai, 2017; Tiwari et al., 2018). According to the recommendations by the previous studies, we selected ANFIS-based model to simulate water quality in the proposed framework. In fact, a control point was selected in the simulated river where a critical point for the aquatic habitats (in the case study, control point was located at downstream of the diversion dam). Due to importance of the point sources of pollution in the case study, normalized total pollutant load by point sources of the water pollutant was applied to generate data driven water quality model in the control point. Long-term water quality data as the recorded data in the control point was utilized in the training and testing process of the data driven model. Some main water quality parameters were selected for water quality assessment including water temperature, dissolved oxygen, phosphate, nitrate and electrical conductivity. More details regarding the responsibilities of each layer have been addressed in the literature (Awan & Bae, 2014). Hybrid algorithm was utilized for training process in all the ANFIS based models in the present study.

Selecting the correct inputs in the ANFIS based models is crucial to develop a robust water quality model. Based on the initial assessment, three inputs should be considered in the data-driven model to simulate dissolved oxygen, phosphate, nitrate and electrical conductivity including normalized pollutant load, stream temperature and rate of river flow. Concentration of the constituents in the river might be altered by changing the stream temperature and rate of flow. Hence, selecting these two parameters is logical in the data-driven model. Moreover, normalized pollutant load at upstream of the control point might help the data-driven model to simulate concentration of the constituents correctly. Thus, we added this parameter as the third input of the model. Table 1 displays more details regarding water quality models.

**Table 1:** Main characteristics of the data driven model for water quality parameters including dissolved oxygen, phosphate, nitrate and electrical conductivity

Inputs	Number of membership functions (inputs)	Type of membership functions (inputs)	Outputs	Number of membership functions (output)	Type of membership functions (output)	Clustering method
Flow rate (m^3^/s)	5	Triangular	Concentration for each water quality parameters including dissolved oxygen, phosphate, nitrate and electrical conductivity	5	Triangular	Subtractive Clustering
Water temperature (°C)	5	Triangular				
Normalized total load(between zero and one)	5	Triangular				

Simulation of water temperature needs a standalone model. Different parameters might affect the stream temperature. However, rate of flow, air temperature and top width of the river channel might be the most effective parameters in the simulation of stream temperature. Hence, we used these parameters as the inputs in the ANFIS based model of the stream temperature. More details regarding stream temperature data driven model is displayed in the Table 2.

**Table 2:** Main characteristics of the ANFIS based stream temperature model

Inputs	Number of membership functions (inputs)	Type of membership functions (inputs)	Outputs	Number of membership functions (output)	Type of MFs (output)	Clustering method
Flow rate (m3/s)	5	Triangular	Water temperature at the control point	5	Triangular	Subtractive Clustering
Wetted perimeter(m)	5	Triangular				
Air temperature (°C)	5	Triangular				

It is required to measure the robustness of the data driven models. Two indices were utilized in this regard including the Nash–Sutcliffe model efficiency coefficient (NSE) and root mean square error (RMSE) as displayed in the following equations where OBS is observed data and SIM is the simulated data.

1 $$NSE=1-\frac{\sum_{t=1}^{T}{({OBS}_{t}-{SIM}_{t})}^{2}}{\sum_{t=1}^{T}{({OBS}_{t}-OBSm)}^{2}}$$

2 $$RMSE=\sqrt{\frac{\sum_{t=1}^{T}({{OBS}_{t}-{SIM}_{t})}^{2}}{T}}$$

### Physical habitat simulation block (Fish habitats)

**Fig. 2:**
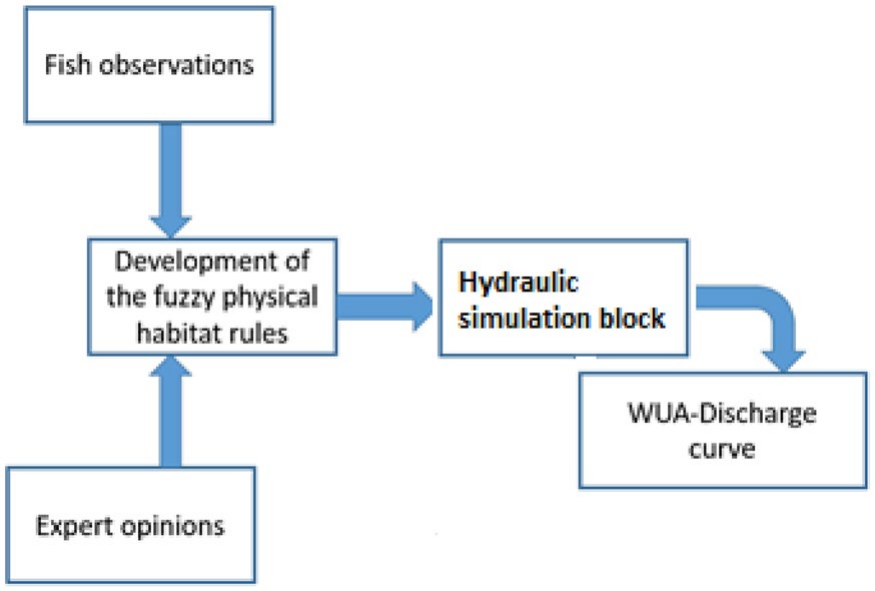
Flowchart of the physical habitat simulation in the proposed framework

Physical habitat suitability has been highlighted as one of the most important factors to sustain the ecological status of the river. Thus, it was considered as one of the blocks in the developed method. Figure 2 displays flowchart of the fuzzy physical habitat simulation presented in the introduction in which combination of the fish observations and expert opinions might be used to develop verbal fuzzy rules of the physical habitat suitability. Then, developed rules will be combined with the results of hydraulic simulation block that has been described. The fish observations were carried out in the representative reach with length of 1000 m at downstream of diversion dam in the case study. The electrofishing method as one of the reliable indirect method for observing fish was applied. Moreover, hydraulic characteristics including depth, velocity and substrate were measured in the sampling points as well. More details regarding methodology of the field studies have been addressed in the literature (Harby et al., 2004). It should be noted that the selected representative reach was used for all the physical simulations. WUA means weighted useable area which is the main output of physical habitat modelling. It should be graphed in respect to river flow (discharge) to show how changing flow is effective on the suitability of habitats. More details have been addressed in the literature (Harby et al., 2004; Sedighkia et al., 2021b).

### Macroinvertebrates population simulation block

Development of the macroinvertebrate response model needs some steps. In the first step, field study is essential. Surber sampler was used to sample macroinvertebrates in the actual habitats. This tool is one of the most applicable tools to take quantitative samples of the organisms that live in the sediment or gravel of the stream bed. Its area was 900 cm^2^ (30 cm*30 cm) with 250 micro-meter mesh size. Sampling was carried out in the different seasons. More details on the sampling method by the Surber sampler has been addressed in the literature (e.g., Doretto et al., 2020). Observed data will be shown in the results of the study. Total amount of benthos in each sampling point was submitted for developing the data driven model of the macroinvertebrate response. Moreover, portable water quality device was utilized to measure water quality parameters as presented in the previous section. Simultaneous sampling and recording were considered in the field studies. In the next step, all the measured water quality parameters in each point were applied as the inputs for the model. Moreover, population of the macroinvertebrates was considered as the output of the model. Table 3 displays more details regarding ANFIS based model of the macroinvertebrate response. RMSE and NSE were applied to measure the performance of the model like the water quality model.

**Table 3:** Main characteristics of the ANFIS based macroinvertebrate response model

Inputs	Number of MFs (inputs)	Type of MFs (inputs)	Outputs	Number of MFs (output)	Type of MFs (output)	Clustering method
DO (mg/L)	5	Triangular	Normalized population of the macroinvertebrates (%)	5	Triangular	Subtractive Clustering
Nitrate (mg/L)	5	Triangular				
water temperature (°C)	5	Triangular				
Phosphate (mg/L)	5	Triangular				
EC(us/cm)	5	Triangular				

### Macrophytes habitat suitability block

**Fig. 3:**
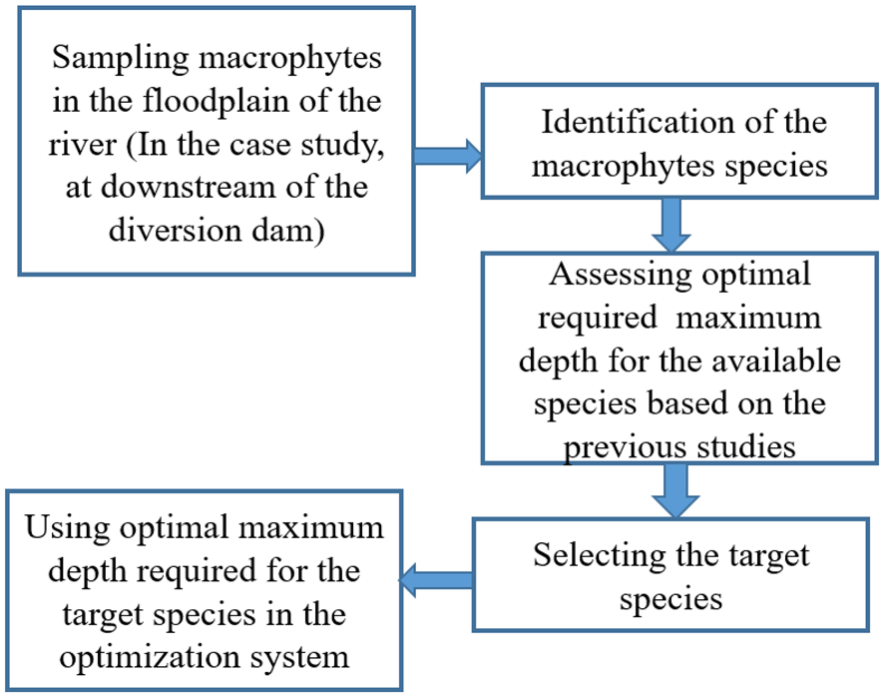
flowchart of the physical habitat suitability for macrophytes in the proposed framework

Building block methodology (BBM) presented in the introduction has recommended to consider aquatic vegetations in the assessment of the environmental flow regime. In fact, not only the fishes and macroinvertebrates require suitable habitats, but also aquatic vegetations need suitable habitats for increasing ecological sustainability in the river ecosystems. Thus, we highlighted aquatic vegetations in the proposed framework as well. Macrophytes are aquatic plants growing in or near water that are one of the important organisms in the river ecosystems. This species are strongly dependent on the physical properties of the river flows. It should be noted that water has a much higher density compared with the air. Thus, mechanical stresses might be a challenge for the macrophytes that could be observed in the floodplains of the rivers. In fact, an appropriate environmental flow regime should be able to mitigate mechanical stresses for the macrophytes in the floodplain of the rivers. We focused on the physical habitat suitability of the macrophytes that has been reviewed as the ecohydraulic response of the macrophytes to the change of the environmental flow. Previous studies corroborate that the maximum depth in the floodplain is a key physical parameter for the macrophytes (Maddock et al., 2013). Hence, we focused on the maximum depth in the simulation of the physical habitat suitability for the macrophytes. Figure 3 displays the proposed workflow of the assessment of the physical habitat suitability for the macrophytes that could be utilized in the structure of the environmental flow optimization.

### Geomorphological suitability block (Spawning habitat suitability)

**Fig. 4:**
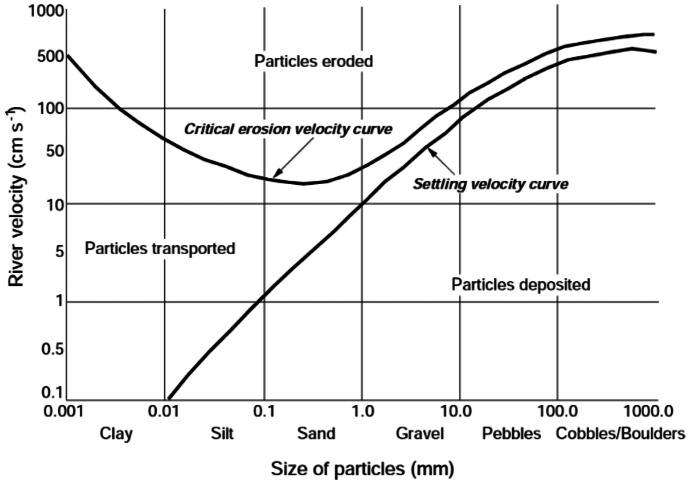
Hjulstrum graph for sediment transport assessment proposed by de Villiers et al. (2008)

**Fig. 5:**
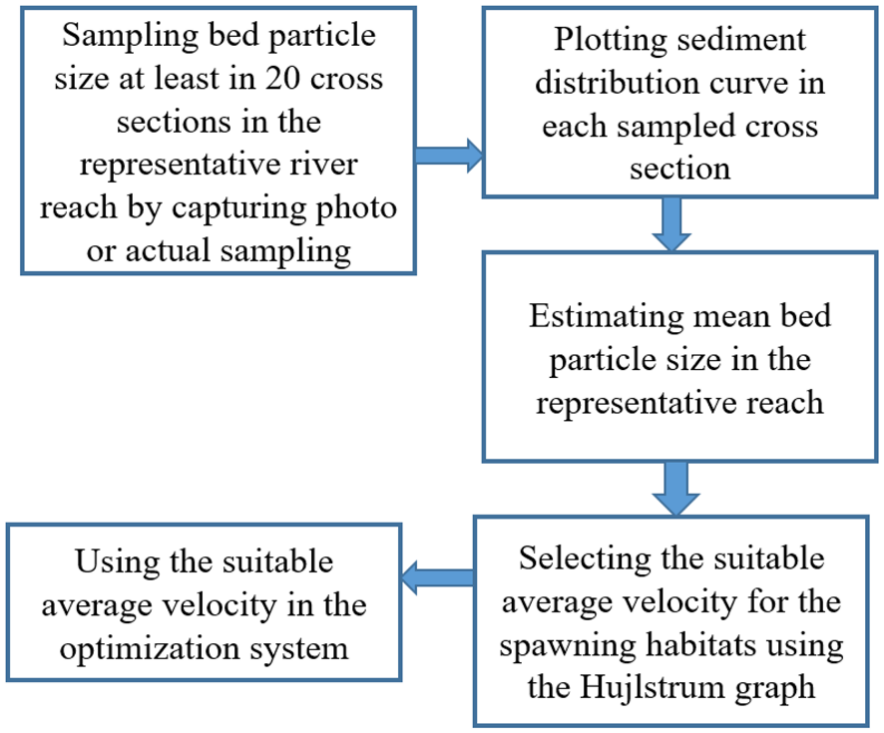
Flowchart of the geomorphological spawning habitat suitability in the proposed framework

**Fig. 6:**
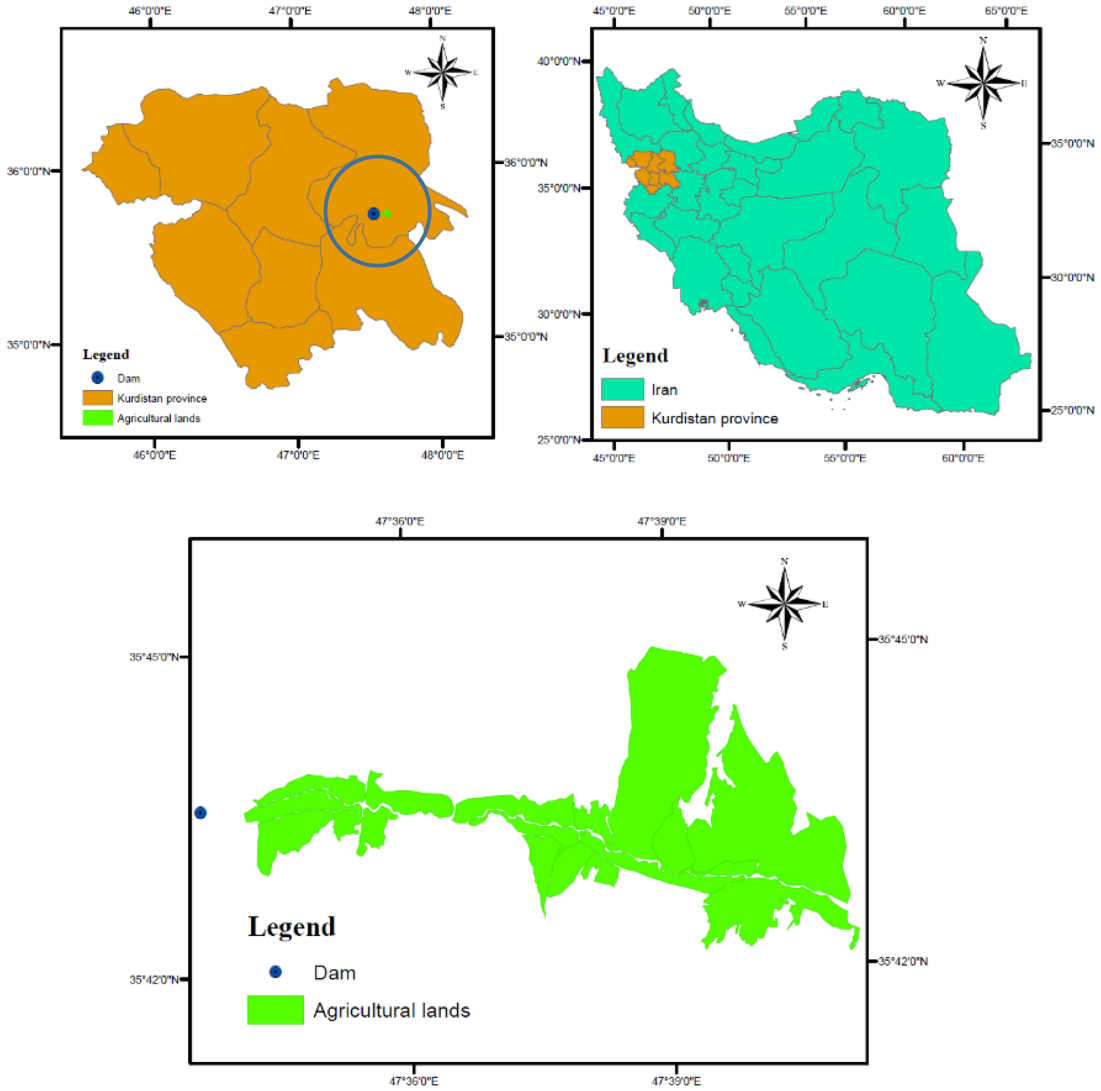
Location of study area and other essential details

Geomorphology of the rivers might be effective on the assessment of the environmental flow as well. Hence, we considered a block for the geomorphological suitability in the proposed method. It should be noted that many geomorphological effects might be possible due to changing the environmental flow in the rivers. However, it might not be possible to come to picture all the effects in one framework or it might make the process of the environmental flow assessment too complex. Hence, the proposed method focused on one of the most important aspects for the geomorphological suitability in the river ecosystems. The reproduction is a critical biological activity that is vital for survival of the aquatic generations in the river ecosystem. As reviewed, the fishes are a good ecological index to assess ecological suitability in the river ecosystems. The process of the fish reproductions needs a suitable bed that is dependent on the river flow. In fact, many fishes immigrate to the upstream of the river for the reproduction to put the eggs in the redds. Erosion and deposition might be happened due to sediment transport in the form of bed load or suspended load in the rivers. Each cross section of the river might experience sediment scour, deposition, or both. According to the literature, If the depth of scour (D_erosion_) is greater than the depth of the redd (D_redds_), the eggs will be washed or the eggs will be lost. Moreover, if the depth of deposition (Ddeposition) is greater than the depth of the redd (D_redds_), the eggs might be lost due to inability of the larva for swimming out of the gravel bed. Hence, deposition and erosion might not be suitable for survival of the eggs in the river habitats (Glawdel, 2011; Naghibi et al., 2011). It should be noted that erosion and deposition could not be zero in practice for all the seasons. Each fish species might reproduce in a certain time of the years. Thus, it might be proper to minimize erosion and deposition in the river in the assessment of environmental flow regime. BBM recommended using the Hjulstrum graph in the assessment of the geomorphological suitability in the rivers as displayed in the Fig. 4 (de Villiers et al., 2008). As could be observed, the zones are classified by this graph including erosion zone, deposition zone and transition zone. In the erosion zone, eroding the bed river is probable due to the bed load transport. In the deposition zone, scouring the bed particles might be occurred. In contrast, no erosion or deposition is not predictable in the transition zone. However, the sediment transport from the upstream is possible in this zone. The Hjulstrum graph (Fig. 6) generates a relationship between flow velocity and bed particle size that means an optimal flow velocity is needed in the different cross sections to minimize egg loss. The Fig. 5 displays the workflow of the spawning habitat suitability in the present study.

### Ground water level simulation block

We applied the ANFIS-based model to simulate ground water level (GWL) in the study area to minimize GWL deterioration in the optimization model. We defined inputs in the data driven model based on the most important effective factors for changing GWL in the simulated aquifer. Four inputs including monthly pumping rate of water from the wells (MCM), monthly irrigation returns flow (MCM), mean rainfall recharge (mm) in the time step t and GWL in the time step t-1. In the case study, monthly irrigation returns flow and mean rainfall recharge were estimated by regional water authority. The output of the model is GWL in the time step t. Each data-driven model needs some indices to measure the robustness of the model. Other characteristics of the GWL data driven model is the same with the water quality data driven models.

### Hydrological analysis block

The purpose of the hydrological analysis in the proposed framework is to assess mean monthly flow in the dry years, normal years and wet years. In fact, the proposed method assesses the environmental flow regime in three hydrological conditions including dry years, normal years and wet years. Stream drought index (SDI) was applied as a known index to assess drought in the case study. At the first step, it is essential to provide time series of monthly flow. Then cumulative stream flow volume should be calculated as displayed in the Eq. (3):

3 $$V_{i,k}={\textstyle\sum_{j=1}^{3k}}Q_{i,j}\;i=1,2,\dots,12\quad k=1,2,3,4$$

where K means period of drought analysis (three to twelve months). In the next step, it is needed to use Eq. (4) to compute SDI.

4 $${SDI}_{i,k}=\frac{{v}_{i,k}-{V}_{k}}{{S}_{K}}\; i=\mathrm{1,2},\dots ,12\quad k=\mathrm{1,2},\mathrm{3,4}$$

where V and S are mean and standard deviation of cumulative stream flow volume respectively. More details regarding stream drought index (SDI) and quantitative values of the index have been addressed in the literature (Akbari et al., 2015). We utilized 12 months SDI to determine hydrological status in the river including dry years, normal years and wet years. Then, we considered years with status of moderate to extreme drought as dry years, non-drought and mild drought as normal years and other values as wet years.

### Water demand assessment block

Water demand should be assessed based on available water demand in the study area. In some regions, main users of water are farmers who consume water for irrigating the agricultural lands. Moreover, urban water demand or industrial water demand are the main consumers of water in some cases. However, water demand might be needed for all sectors in some case studies. It is needed to analyse water demand in the study area to generate the water demand time series.

### Multiobjective optimization block

According to the requirements for optimizing the environmental flow regime, we proposed a novel multiobjective optimization in which six purposes were considered as displayed in the Fig. 1. Equation (5) displays the objective functions of the optimization model.

5 $$Minimize\left(OFs\right)=\left\{\begin{array}{c}Physical\; habitat\; loss,\; Objective\; 1\\ Benthos\; population\; loss,\; Objective\; 2\\ Macorphytes\; habitat\; loss,\quad Objective\; 3\\ Spawning\; habitat\; loss\qquad\qquad Objective\; 4\\ GWL\; loss\;\qquad\qquad\qquad\qquad Objective\; 5\\ Water\; supply\; loss\qquad\qquad\qquad Objective\; 6\end{array}\right.$$

Equations (6) to (11) show the definition of the losses in the optimization model where OWUA is normalized optimal weighted useable area, OBP is normalized optimal benthos population, SDM is suitable depth for macrophytes, ODM is optimal depth for macrophytes habitat proposed by the optimization model, SVS is suitable velocity for spawning habitats, OVS is optimal velocity for spawning habitats, GWL1 is the ground water level in the step 1 of the simulation period, GWL is optimal ground water level, DWD is defined water demand for the study area and OR is optimal release proposed by the optimization model to the downstream river of diversion dam in the case study.

6 $$Objective\;1=({\textstyle\sum_{t=1}^T}{(\frac{1-{OWUA}_t}1)}^2)/T$$

7 $$Objective\;2=({\textstyle\sum_{t=1}^T}{(\frac{1-{OBP}_t}1)}^2)/T$$

8 $$Objective\;3=({\textstyle\sum_{t=1}^T}{(\frac{SDM-{ODM}_t}{SDM})}^2)/T$$

9 $$Objective\;4=({\textstyle\sum_{t=1}^T}{(\frac{SVS-{OVS}_t}{SVS})}^2)/T$$

10 $$Objective\;5=({\textstyle\sum_{t=1}^T}{(\frac{GWL1-{GWL}_t}{GWL1})}^2)/T$$

11 $$Objective\;6=({\textstyle\sum_{t=1}^T}{(\frac{{DWD}_t-{OR}_t}{{DWD}_t})}^2)/T$$

We applied the multiobjective particle swarm optimization (MOPSO) to find the best solution for the optimization problem. More details regarding MOPSO has been addressed in the literature (Coello et al., 2004). The proper method for selecting the best solution among available non-dominated solutions is a challenging step to finalize the optimal solution for the problem using MOPSO. In the proposed method, the optimal solution was selected based on the minimizing distance between outputs of the objective functions. In fact, the optimal solution might provide a fair balance between losses for river ecosystem, ground water and water supply. Some indices were used to measure the performance of the environmental flow optimization as displayed in the Table 4.

**Table 4:** Indices for measuring the performance of the optimization system

**Index**	**Description**
Mean annual environmental flow (percentage of mean annual flow)	This index measures the ratio of assessed environmental flow to annual available water in the river
Mean annual water supply by surface water and ground water resources (%)	This index measures the ratio of water supply to defined water demand
Mean annual water supply by ground water resources (%)	This index measures the ratio of water supply by surface water (river)
Mean annual water supply by surface water resources (%)	This index measures the ratio of water supply by ground water
Mean normalized weighted useable area (-)	This index indicates the mean normalized WUA in the simulated period
Normalized population of macroinvertebrates (%)	This index indicates the mean normalized population in the simulated period
RMSE for optimal mean velocity in the spawning habitats(m/s)	This index indicates the mean error for providing suitable mean velocity in the spawning habitats
RMSE for optimal maximum depth in the floodplain for macrophytes (m)	This index indicates the mean error for providing suitable maximum depth of the macrophytes habitat
Maximum GWL deterioration (m)	This index indicates the maximum GWL deterioration in the simulated period

### Case study

We implemented the proposed method in one of the river basins in the Kurdistan province, Iran. The main economic activity in this basin is the agriculture and many rural areas are available that means cultivated areas are considerable. In the current condition, the main water resource for supplying irrigation demand for these agricultural lands is ground water and surface water. The total available area of agricultural lands is approximately 64,000 Ha. Many wells could be observed in this region. However, there is a serious concern for regional water authority in terms of GWL deterioration. Thus, it is recommended to supply irrigation demand by the surface water resources where are accessible in the study area. The available river in the study area might be a good option for supplying part of irrigation demand. Hence, a small dam has been constructed in this region. It should be noted that storage capacity in this dam is very limited. Hence, we considered it like a diversion dam in the present study. Water-level data for ground water simulation have been observed in 9 piezometers which have been drilled and are continuously maintained and monitored by the Kurdistan Water Authority. The study area includes 120 production wells. We used mean recorded ground water level by the piezometers to develop data driven model of GWL. It was necessary to assess a suitable environmental flow regime at downstream river of the diversion dam. An integrated optimization framework is needed due to challenges for management of the environmental flow, GWL deterioration and water supply. Figure 6 displays the location of the Kurdistan province and study area. It should be noted that main user of water in the case study are farmers which means irrigation demand was considered in the modelling process. This research work presents a combined framework. Hence, it is helpful to shows the inputs and outputs in the case study clearly. Furthermore, more clarification on calibration and validation of models is needed. Table 5 shows the inputs, outputs and method of calibration and validation of the models in the case study.

**Table 5:** Inputs, outputs and calibration/validation method of models in the case study

Model	Inputs	Outputs	Calibration/validation method
Hydraulic modelling	Digital elevation model, land use map in floodplains, recorded water level and river flow at upstream and downstream	Depth and velocity distribution in different cross sections	Using some measured depth and velocity in cross sections considering roughness as the calibration parameter
Physical habitat modelling	Fish observations, opinions by expert panel and outputs of hydraulic modelling	Fuzzy rules of suitability in the first stage and weighted useable area in the second stage	Due to using fuzzy based model, no further calibration\validation was needed
Water quality modelling	Displayed inputs in Tables 1 and 2 (averages in simulated river reach)	Concentration of water quality constituents and water temperature (averages in simulated river reach)	80% of recorded water quality data was used for training and 20% was used for testing the data driven models
Macro invertebrate’s population modelling	Concentration of water quality constituents and water temperature (averages in simulated river reach)	Normalized population of the macroinvertebrates (%)	80% of sampled population observations were used for training and 20% were used for testing the data driven models
Ground water level modelling	monthly irrigation returns flow (MCM) estimated by regional water authority, mean rainfall recharge (mm) in the time step t estimated by regional water authority and GWL in the time step t-1 recorded in observation wells (averages in the plain)	GWL in the time step t	80% of recorded or estimated data was used for training and 20% was used for testing the data driven model

## Results

### Physical fish habitat simulation

**Fig. 7:**
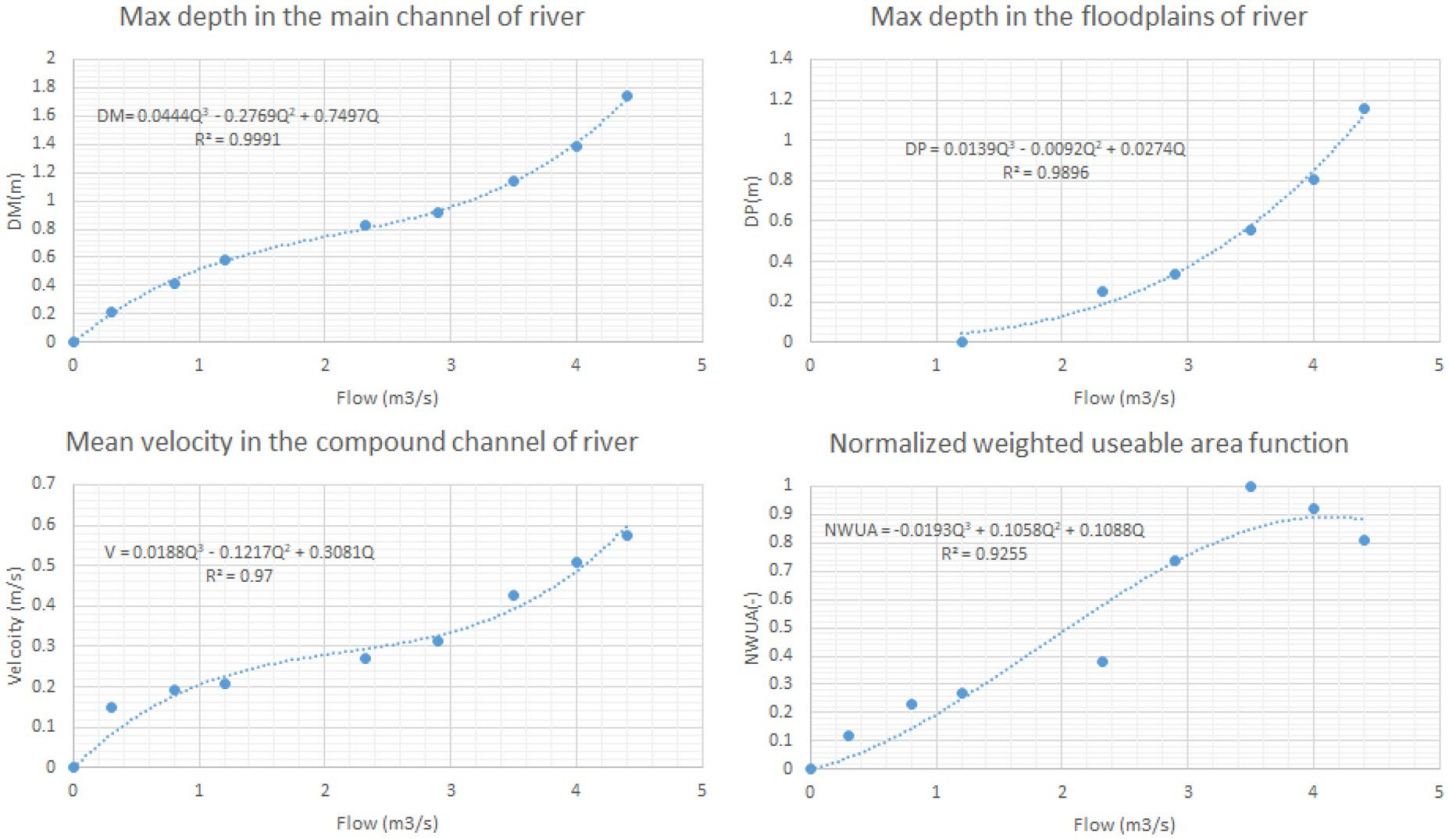
Results of the hydraulic simulation and physical habitat simulation in the representative reach in the case study

In the first step, it is required to present results of the simulations or analysis in the case study based on the SOBM. Figure 7 displays the main outputs of the hydraulic simulation and fuzzy physical habitat simulation blocks. Based on the needs for the next steps of the developed environmental flow regime method, relationship (an average relationship for all the simulated cross sections in the representative reach) between river flow and maximum depth in the main channel and the flood plain of the river are displayed in this figure. Moreover, relationship between river flow and mean velocity in the compound channel of the river is shown as well. Normalized weighted useable area is the final output of the fuzzy physical habitat simulation in the representative reach that could be observed in the Fig. 7. We applied the regression model for finalizing the developed functions that have been utilized in the optimization block.

### Hydrological analysis

**Fig. 8:**
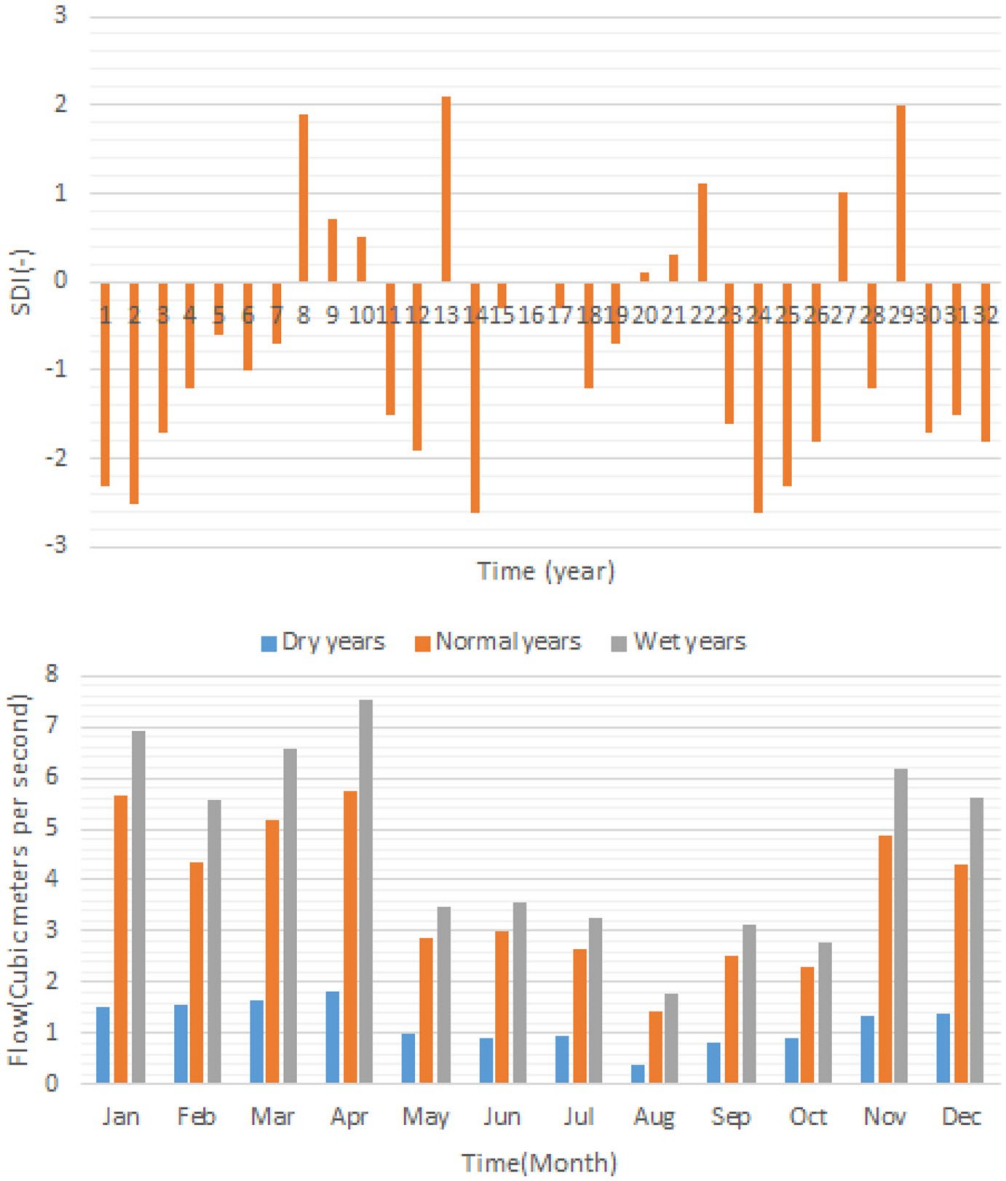
Results of drought analysis and average monthly flows in different hydrological condition

The Fig. 8 displays the result of the hydrological analysis block in which SDI time series in a long-term simulated period is displayed. Moreover, mean monthly flow in the dry years, normal years and wet years have been displayed as well. According to the results, the significant difference between dry years and normal or wet years could be observed. However, the river flows in the normal years and wet years are not very different. It sounds that the management of the river ecosystem in the dry years or severe droughts might be a serious challenge due to lack of sufficient water to supply both water demands and environmental flow regime. Furthermore, air temperature in dry years, normal years and wet years were utilized for the water quality modelling.

### Water quality modelling

**Fig. 9:**
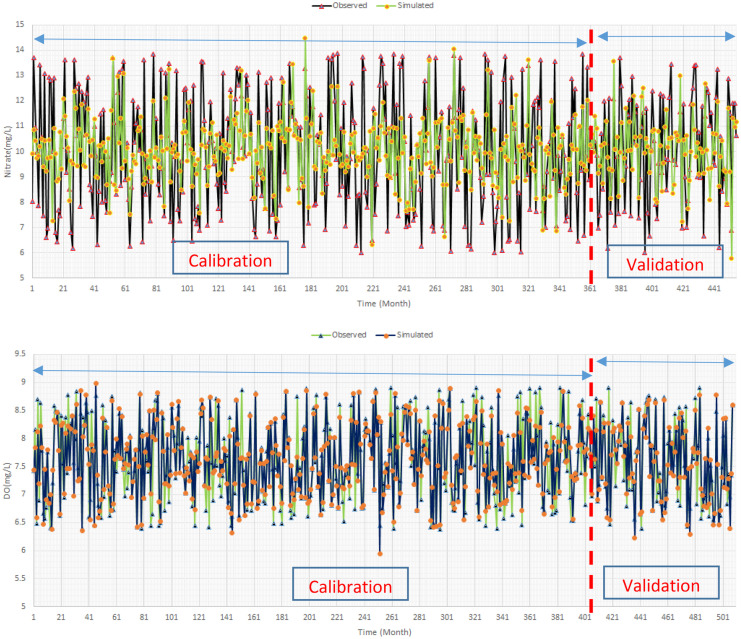
A sample of training and testing process of the water quality models

The next step is to present the training and testing results of the water quality modelling. As presented, the data driven models were applied to simulate the water quality in the case study. Hence, it is required to measure the robustness of the data driven models. Figure 9 displays a sample of the training and testing result of the data driven water quality models in which nitrate and dissolved oxygen (DO) concentration are shown. Two indices including RMSE and NSE were applied to assess the robustness of the data driven models that are displayed in the Table 5. The robustness of the data driven models in terms of water quality parameters is different that should be considered in the uncertainty analysis of the final outputs of the environmental flow optimization. The data-driven model for simulating water temperature is robust. However, models for other water quality parameters might not be as robust as the water temperature model. In fact, we considered some main inputs for simulating the water quality parameters. However, other factors might be effective as well, which are not considered in the simulation block. Hence, developing accurate data-driven water quality model might not be possible in all of the cases. According to the literature, if NSE is more than 0.5, the developed model is very robust in terms of the predictive skills. However, NSE more than 0.3 might be defensible as the acceptable predictive skills for the data driven models. Developed data-driven models are reliable for using in the structure of the optimization model based on Table 6.

**Table 6:** Evaluation indices for water quality models in testing period (validation period)

Model	NSE	RMSE
Stream temperature	0.8	1.04
DO	0.74	0.3
Nitrate	0.36	1.75
Phosphate	0.35	0.13
EC	0.37	149.8

### Macroinvertebrate’s population modelling

**Fig. 10:**
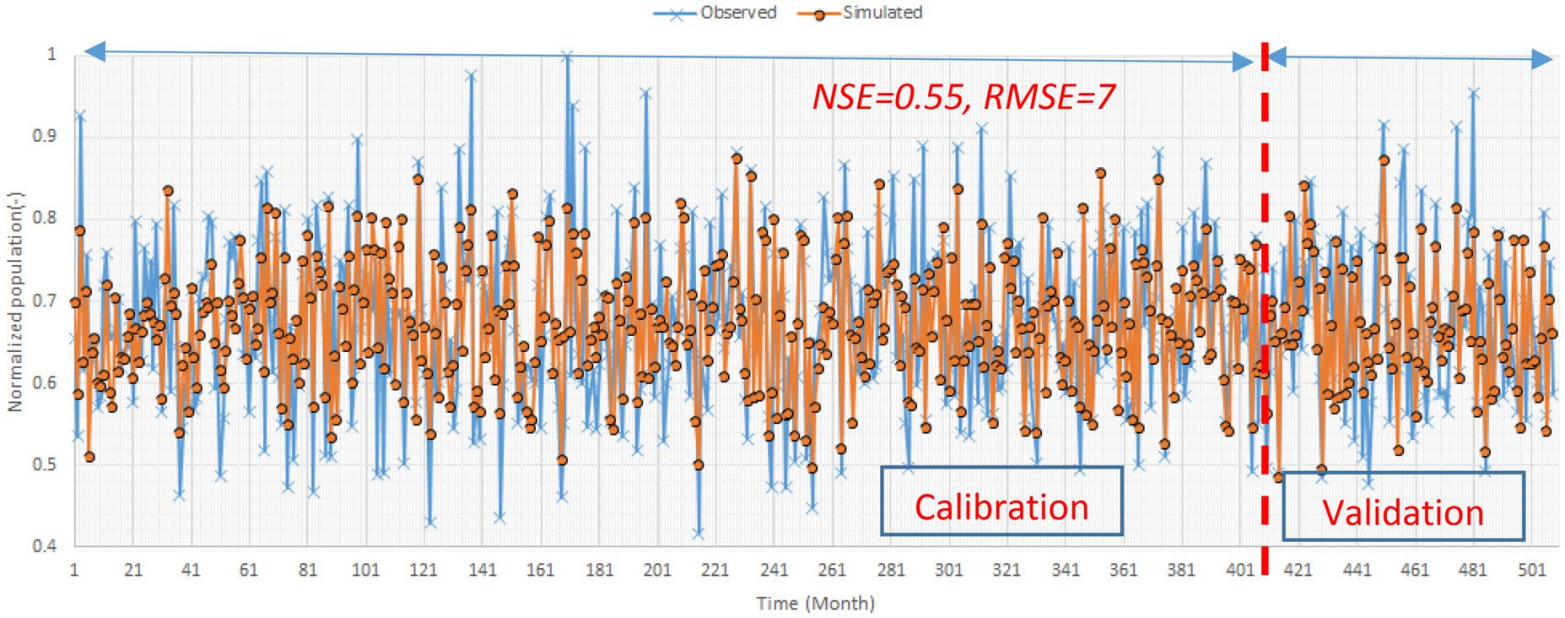
Training and testing process of the macroinvertebrates population model

In the next step, it is essential to present the validation results of the macroinvertebrates population simulation block. Figure 10 displays the training and testing process of this model in which normalized population is the output of the model. We utilized RMSE and NSE to measure the robustness of this model as well as water quality models. According to the results, developed model is reliable to simulate the population of the macroinvertebrates in the simulated river reach.

### Macrophytes habitat assessment

Suitable maximum depth for the target species of the macrophytes was considered 0.2 m based on the proposed method for assessing macrophytes habitat loss. Furthermore, suitable mean suitable velocity for minimizing spawning habitat loss was considered 0.209 m/s based on the developed methodology for spawning habitat suitability.

### Ground water level (GWL) modelling

**Fig. 11:**
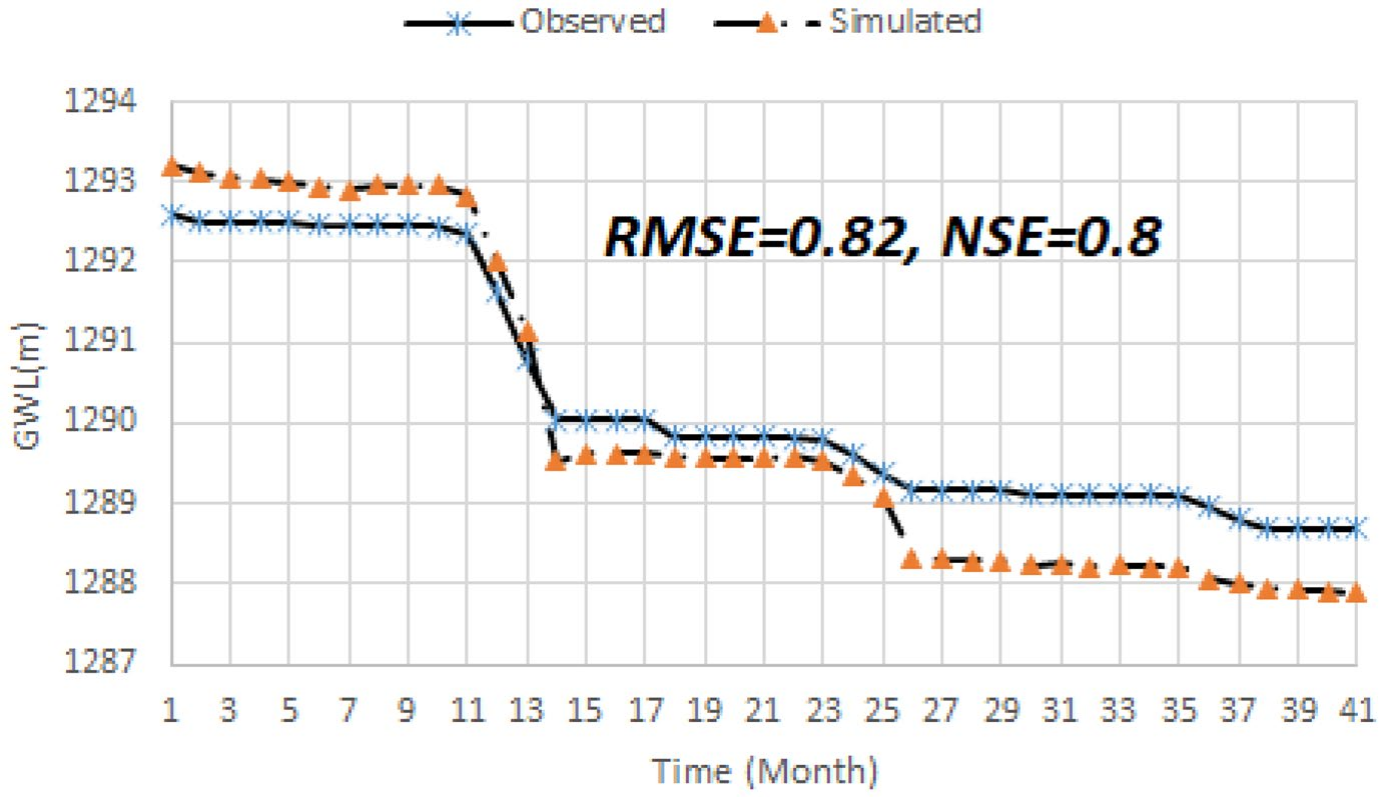
Testing process of the GWL data driven model (validation period)

Another important result is the GWL simulation block that is applied in the optimization block to predict or simulate GWL in the case study. As presented, RMSE and NSE were applied to measure the robustness of this model as well. Figure 11 displays testing results of the GWL model in which two measurement indices are shown. The measurement indices demonstrate that the GWL model is reliable for further applications. In fact, it is able to predict the GWL with the acceptable error in the case study.

### Environmental flow optimization

**Fig. 12:**
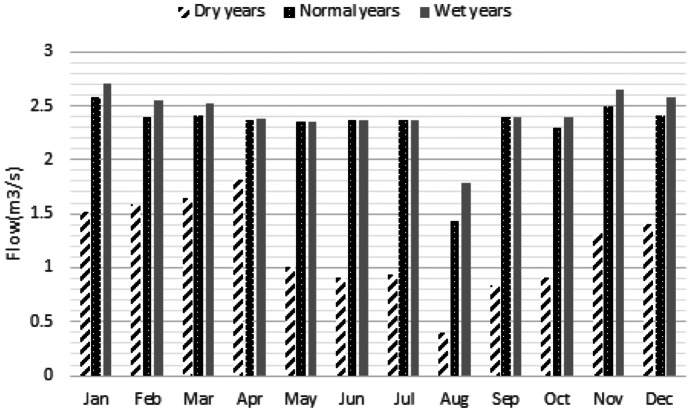
Optimal environmental flow regime proposed by optimization system in the case study

**Fig. 13:**
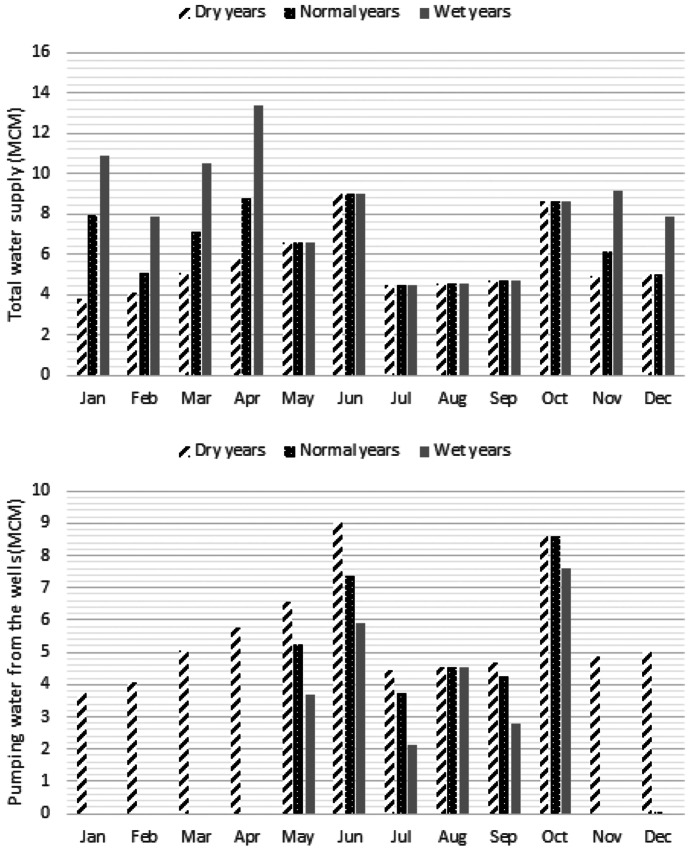
Optimal water supply proposed by optimization system in the case study

In the next step, results of the optimization block should be presented as the main outputs of the developed framework in which optimal environmental flow regime could be finalized. Figure 12 displays the optimal environmental flow regime in dry years, normal years and wet years. Difference of normal years and wet years in terms of environmental flow is not considerable in some months. However, their difference is not negligible in some other months. It should be noted that difference between dry years and normal years is significant in terms of optimal environmental flow. Figure 13 displays optimal water supply by the proposed method in which total water supply and water supply by the groundwater resources are shown. The results indicate that optimal water supply due to changing hydrological condition might be different. The optimization model is able to supply part of the defined water demands. It is observable that water demand in dry years is totally supplied by the groundwater resources. In fact, there is not sufficient water in the river for dry years that means supplying water demand should be carried out using the wells. In fact, offstream flow in the dry years is close to zero.

**Fig. 14:**
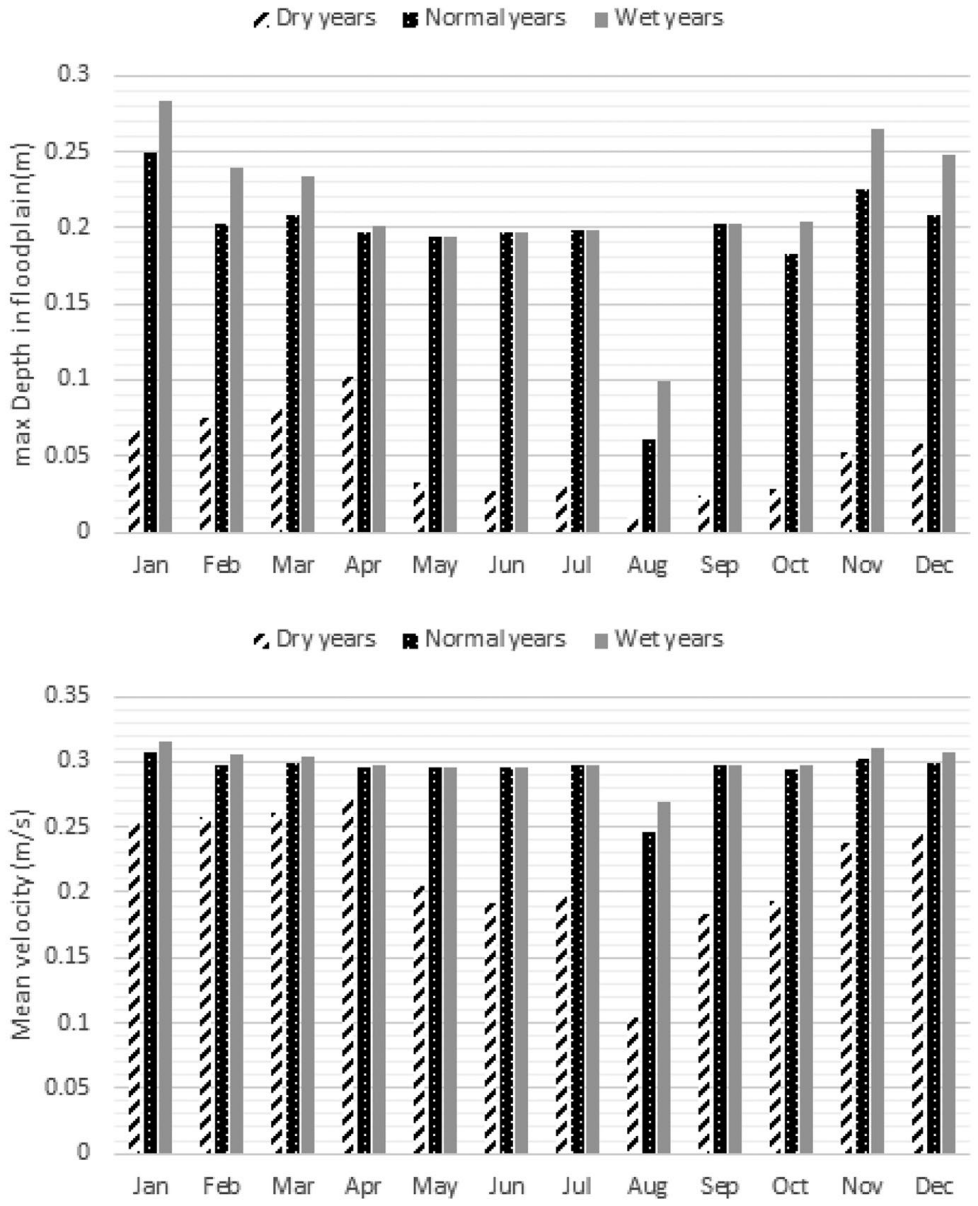
Optimal maximum depth in the flood plains and mean velocity in the compound channel of the representative reach proposed by optimization system in the case study

**Fig. 15:**
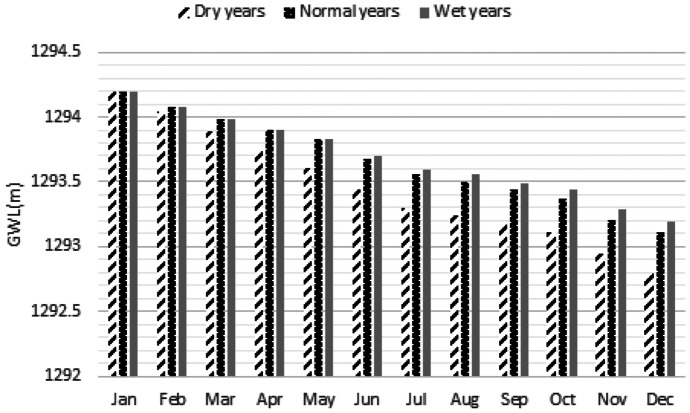
Optimal ground water level proposed by optimization system in the case study

**Fig. 16:**
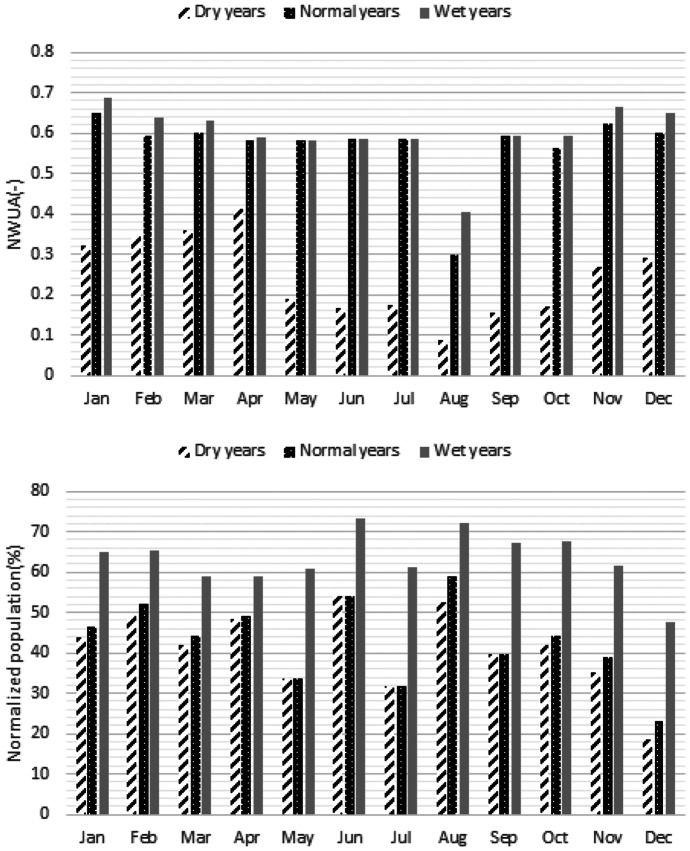
Optimal NWUA and normalized macroinvertebrates population proposed by optimization system in the case study

Figure 14 displays the optimal maximum depth in the flood plains and mean velocity in the compound channel of the river that indicates the severe droughts might be very destructive for the macrophytes due to significant reduction of the maximum depth in the flood plains. More discussion will be presented in the next section. Figure 15 shows GWL in the simulated periods that demonstrates significant reduction of the GWL especially in the dry years might be inevitable. Moreover, Fig. 16 displays normalized weighted useable area and normalized population of the macroinvertebrates in different hydrological conditions. Performance of the optimization model is not similar in terms of these ecological aspects. For example, normalized weighted useable area in the dry years is considerably less than normal years and wet years. It might be related to the lack of sufficient environmental flow in the river. In fact, suitability of the physical habitats is not naturally appropriate. In other words, considering supply of water demand by the river in the dry years is drastically destructive for the river ecosystem. Hence, the optimization model made the offstream flow zero to minimize ecological degradation in the droughts. Conversely, the normalized population in the dry years and normal years are similar. However, it is increased in the wet years remarkably. In fact, other factors such as air temperature might change the role of environmental flow on the normalized population of the macroinvertebrates in the simulated river reach. Measurement indices of the optimization system will be discussed in the next section.

## Discussion

Table 7 displays the measurement indices for the case study. According to this table, mean annual environmental flow (percentage of mean annual flow) is 100%, 62% and 52% in the dry years, normal years and wet years respectively. In fact, it is critical not to use the available water in the river for the dry years. In other words, no water abstraction for agriculture from river ecosystem in dry years is possible. Old methods such as hydrological desktop methods underestimate the environmental flow. For example, Tennant method proposed 10% of MAF as the minimum environmental flow that might not be proper in many cases. Thus, avoiding methods in which ecological assessment are not highlighted is vital in the assessment of the environmental flow. In fact, underestimation or overestimation by these methods might raise either ecological impacts or water supply loss. Mean annual water supply demonstrates that reducing water demand is necessary in the case study. However, the role of the groundwater is diminished significantly in the wet years. Hence, the optimization model is able to balance the role of surface water and groundwater for supplying demands fairly. Mean NWUA is another measurement index that might be useable for better management of river basin. Management of the physical habitats is a serious challenge in the dry years. In fact, remarkable increasing physical habitat loss might be a threat for the river habitats that should be considered in the river basin management.

**Table 7:** Measurement indices for the optimization system of environmental flow regime

Index	Dry years	Normal years	Wet years
Mean annual environmental flow (percentage of mean annual flow)	100.00	62.14	51.53
Mean annual water supply by surface water and ground water resources (%)	58.78	68.97	86.36
Mean annual water supply by ground water resources (%)	100.00	43.44	27.32
Mean annual water supply by surface water resources (%)	0.00	56.56	72.68
Mean normalized weighted useable area (-)	0.25	0.57	0.60
Mean normalized population of macroinvertebrates (%)	40.88	42.99	63.26
RMSE for optimal mean velocity in the spawning habitats (m/s)	0.05	0.09	0.09
RMSE for optimal max depth in the floodplain for macrophytes (m)	0.15	0.04	0.05
Maximum GWL deterioration (m)	1.41	1.09	1.01

Mean normalized population of the macroinvertebrates demonstrates that proposed environmental flow regime is able to balance the population in different hydrological conditions. However, reducing total pollutant load in the simulated river basin is recommendable that might increase the population. RMSE of optimal maximum depth in the floodplain indicates that droughts might be a serious menace for macrophytes, because sufficient depth is not available in the floodplains. However, the physical habitat suitability of the macrophytes habitats is acceptable in the normal years and wet years. Maximum GWL deterioration in the study area indicates that water demand is considerable in the river basin. It is recommendable to reduce water demands by implementable strategies for dwindling destructions of the aquifer and the river ecosystem. In the current condition, GWL deterioration in the dry years is 40% more than wet years due to lack of sufficient available water in the river. The performance of the optimization model is acceptable for balancing the water supply by the surface water and ground water resources. It should be noted that we assumed a proper GWL in the first time step of the simulation.

Each optimization system might have some strength, drawback and limitations that should be noticed by the engineers. Moreover, the future research fields should be discussed as well. The non-linear relationship between river flow and the biological response in fish habitats demonstrates that using ecological based models in the assessment of the environmental flow is necessary. In fact, old methods of the environmental flow regime considered a linear relationship between the biological response of the aquatics and the river flow that might create a misconception regarding the assessment of environmental flow. The non-ecological based methods for assessing environmental flow should be excluded in the future studies. In other words, they are not reliable regarding the assessment of the correct environmental flow regime. Unfortunately, many recent studies in the water resource optimization utilized the simple environmental flow methods. The outputs of the present study indicate that using an integrated framework such as current framework is reliable for using in the water resource management of the river basins in which GWL is manageable as well.

Several simulation blocks were developed in the proposed framework that were able to carry out responsibilities properly. However, other alternatives might be available as the simulation methods that might be highlighted in the future studies. For example, using data driven models in the simulation of physical habitat have been recommended in the literature as well. They might generate reliable results in some cases. Fuzzy physical habitat simulation was proposed in the present study due to flexibility and robustness. Moreover, using hydrodynamic models to simulate water quality might be helpful as well. These models could not be used in the structure of the optimization model directly. However, they might be useful to develop a more robust data driven model when sufficient recoded data is not available. Furthermore, a simple model was used to simulate the physical habitat suitability of the macrophytes in the floodplain that might not be appropriate in all the cases. Hence, improving the macrophytes habitat suitability model is recommendable in the future studies. The simulation of GWL is another aspect in the simulation process of the proposed method. A specific form of the ANFIS based model was applied in the present study. However, other data driven models might be utilizable in this regard based on the technical consideration in the case study. More details regarding the available machine learning models to simulate GWL have been addressed in the literature.

The sources of uncertainties in the proposed framework should be discussed as well. The first source of the uncertainties is field studies. In fact, lack of sufficient data or incorrect data might be problematic to generate a correct environmental flow regime. The engineers should try to minimize these uncertainties using modern tools and several measurements. The second source of the uncertainties in the proposed method is unreliability of the data driven models. Two options might be recommendable in this regard including utilizing the robust machine learning methods and a wide range of the recorded data to develop the data driven model. Finally, computational limitations of the optimization algorithms is another source of the uncertainties in the developed model that will be discussed.

The main problem of using metaheuristic optimization is inability of these algorithms to guarantee the global optimization. Thus, it might be recommendable to apply different algorithms for finalizing the optimal solution by a decision-making system. It should be noted that limited number of multiobjective algorithms have been developed in the literature. Hence, changing the proposed objective functions to a single function might be helpful due to availability of many single objective algorithms in the literature. Many new generation algorithms such as bat algorithm have been developed in recent years. The computational complexities are another challenge for using the optimization algorithms in the environmental flow assessment. As an official definition on the computational complexities, it might be defined as the required time and memory to find the optimal solution by the optimization algorithm. In fact, high computational complexities might reduce the efficiency of the optimization algorithms. It should be noted that modellers and engineers are not willing to apply optimization models in which computational complexities are high that means needed time and memory might be a barrier for having a successful application of the optimization algorithms. Numerous simulations or covering a long-term period might be essential in the projects. High computational complexities is a serious limitation for the proposed method. Two main sources for computational complexities of the proposed method should be noted including using a multiobjective optimization algorithm and several data driven models in the structure of the optimization algorithms. In fact, the optimization algorithm must open the data driven models in each time step that increases running time and needed memory. One of the main research fields for improving the proposed framework is to minimize computational complexities. For example, changing the optimization algorithms in the form of the single objective optimization might be useful for reducing computational complexities.

Discussion on utility of this research work and technical limitations and future scopes is helpful for the readers in further applications of this study. The proposed method is advantageous for reducing environmental degradations in water resources management in the catchment scale. In fact, it is able to integrate ecological requirements and water resources planning in one model. We considered a wide range of effective parameters in this framework. However, no model is perfect which means many improvements could be added in future studies. Water quality of ground water has not been considered in this study because it was not a critical issue in the case study. However, it should be added in some case studies. Thus, it is recommendable to add the water quality modelling of ground water to the scope of the framework in future works. Furthermore, some case studies might have other types of aquatics which should be added to the environmental flow model. Another component which should be added in some cases is planning of urban water demand.

## Conclusions

The present study developed a novel method for optimizing the environmental flow regime in the rivers in which several simulation blocks and optimization block were applied in an integrated framework. The blocks include hydraulic simulation by HEC-RAS 1D, water quality modelling by ANFIS-based model, physical fish habitat simulation by fuzzy approach, macro invertebrate’s population simulation by ANFIS-based model, macrophytes habitat suitability assessment, geomorphological suitability assessment, GWL simulation by ANFIS-based model, water demand assessment and hydrological analysis by drought analysis method. The proposed model was implemented in a catchment as the case study in which significant water was needed for irrigation the lands. Based on the results, ANFIS-based model is robust for simulating key factors such as water quality and macroinvertebrate’s population. Optimization model is able to satisfy 69% of water demand in normal years, while ecological impacts on the river ecosystem is minimized. Moreover, deterioration of ground water level is mitigated as well. The proposed method is advantageous for reducing environmental degradations in water resources management in the catchment scale. In fact, it is able to integrate ecological requirements and water resources planning in one model.

## Data Availability

The datasets generated during and/or analysed during the current study are not publicly available due to requirements by the data provider but are available from the corresponding author on reasonable request. However, it is not free of charge.
